# Associations between facial emotion recognition and young adolescents’ behaviors in bullying

**DOI:** 10.1371/journal.pone.0188062

**Published:** 2017-11-13

**Authors:** Tiziana Pozzoli, Gianluca Gini, Gianmarco Altoè

**Affiliations:** Department of Developmental and Social Psychology, University of Padua, Padua, Italy; Universita degli Studi di Udine, ITALY

## Abstract

This study investigated whether different behaviors young adolescents can act during bullying episodes were associated with their ability to recognize morphed facial expressions of the six basic emotions, expressed at high and low intensity. The sample included 117 middle-school students (45.3% girls; mean age = 12.4 years) who filled in a peer nomination questionnaire and individually performed a computerized emotion recognition task. Bayesian generalized mixed-effects models showed a complex picture, in which type and intensity of emotions, students’ behavior and gender interacted in explaining recognition accuracy. Results were discussed with a particular focus on negative emotions and suggesting a “neutral” nature of emotion recognition ability, which does not necessarily lead to moral behavior but can also be used for pursuing immoral goals.

## Introduction

The ability to recognize emotional facial expressions is important for everyday interpersonal relationships and for social adjustment [1–2]. Indeed, past research involving children and adolescents showed that accurate recognition of emotions is associated with higher social and academic competence, and with less externalizing and internalizing behaviors ([1,3–5]; see [2] for a meta-analysis).

Quite surprisingly, this basic ability has almost been overlooked in three decades of bullying research, despite the fact that scholars have repeatedly stressed the importance of emotions in this phenomenon (e.g., [6–8]). In particular, in the emotional domain, bullying research has widely focused on empathy, as recently summarized in a systematic review [8] and in two meta-analytic studies [9–10]. However, although these studies showed that understanding and sharing other people’s emotions is related to behavior students’ decide to act during bullying episodes, to date only two studies specifically investigated a more basic skill, that is, emotion recognition ability [11–12].

These two studies certainly have merits and we built on them to develop the present work. Woods and colleagues [12] found that, controlling for gender, peer-nominated bullies did not differ from students not involved in bullying in their ability to recognize emotions, whereas victims scored lower both in the overall ability to identify emotions and, in particular, in recognition of anger and fear. In contrast, Ciucci and colleagues [11] recently found no significant relation between self-reported bullying and victimization and emotion recognition abilities. However, both studies only focused on bullying and victimization, neglecting the social nature of bullying [13]. Indeed, they compared students who bully or who are victims with a general, rather vague category of “uninvolved” students, which could lead to spurious results. Non-aggressive bystanders are not a homogenous category, but quite different behaviors can be adopted by witnesses of bullying (i.e., defending or passive bystanding; [14]) and different bystanders’ behavior are associated with different individual characteristics (e.g., [15–17]).

Methodologically, both studies assessed emotion recognition through static photographs [18–19], which have been suggested to resemble facial expression in everyday communication to a lesser extent than dynamically morphed facial expressions [20–21]. Furthermore, movement is acknowledged to play an important role in emotion perception affecting recognition accuracy [22]. Additionally, both studies investigated four basic emotions (i.e., happiness, sadness, fear, anger) and the rationale for excluding the other two basic emotions (surprise and disgust) was not provided. Finally, intensity of emotions was not considered. However, in real life, emotions are not always full-blown expressed and it has been suggested that presenting facial expressions at both lower and higher intensities could detect more subtle performance differences [21,23].

### The current study

The novelty of the current study is to investigate the relations between four different behaviors in bullying, namely bullying others, being victimized, defending the victim, and passive bystanding, and young adolescents’ ability to recognize facial expressions of the six basic emotions [24], dynamically expressed at different intensities. Although our theoretical interest was focused on participants’ recognition of the negative emotions, which are more likely to characterize bullying episodes (e.g., sadness and fear of victims, disgust and anger of bullies), happiness and surprise were also considered. This allowed us to detect whether observed performances were limited to specific emotions or could be described as a more general (in)ability. Moreover, this may provide other researchers with a more complete picture useful as a basis for future studies.

At a general level, in the whole sample we expected to replicate previous findings about differences in recognition accuracy depending on specific emotions (e.g., [21]), that is, that some emotions (i.e., happiness and anger) are more easily recognized than others (i.e., fear and sadness). Concerning the main goal of this study and given the paucity of studies on this topic in the bullying field, we deemed not appropriate to formulate specific hypotheses on the relation between the recognition of each emotion, at both intensities, and each bullying-related behavior. However, on the basis of both findings in other fields of research (e.g., bystanders’ intervention in social psychology) and previous results in the bullying literature involving other constructs, such as social-emotional skills, some anticipations can be made.

First, consistent with the view of bullies as “competent” individuals [25–27], it is not surprising that, in general, previous studies did not find particular deficits in emotion recognition in youth who bully. However, some differences may be expected considering different emotions at different intensities. We hypothesized that higher levels of bullying could be related with higher ability in detecting fear, which may be a useful means to identify more vulnerable victims within the group and, subsequently, to recognize—and even maximize—the “success” of the aggression. Second, regarding victimization, we expected to replicate previous findings both in bullying and in other fields of research, showing general difficulties in recognizing emotions (e.g., [12, 28]). In particular disgust and anger, if correctly identified, especially when they are not yet full-blown expressed, could be used to predict bullies' attacks and, potentially, avoid them. Third, in line with research that showed that emotion recognition abilities correlate with empathic skills and prosocial behavior [1], we hypothesized that defending behavior would be associated with higher emotion recognition accuracy. This hypothesis also stems from the picture of defenders as socially and emotionally competent [15, 29]. Moreover, given that classic research on bystander intervention suggested that the recognition of target’s distress predicts the likelihood of helping [30–31], a link between recognition of sadness and fear with defending (positively) and passive bystanding (negatively) behavior was expected. Associations in the same directions were hypothesized for disgust and anger. Indeed, recognizing these emotions, for example in the bully’s face, can help to distinguish playful from intentional aggressive behaviors and could, therefore, represent one of the first steps for deciding to intervene in a potentially risky situation like bullying [32].

Finally, literature extensively showed that gender might affect both students’ behavior during bullying episodes [17, 33–34] and emotion recognition abilities [35–36]. Therefore, even though full exploration of gender differences was not the focus of the current study, participants’ gender was considered both as a control variable and a potential moderator in the analyses.

## Method

The research project has been approved by the Ethical Committee for the Psychological Research of the University of Padova (number 17–2151).

### Participants

Participants were recruited from one middle school (6^th^ to 8^th^ grade) located in a medium sized city in the North of Italy. Of 129 students invited to participate, 127 (98.4%) obtained written parental consent. However, due to school absences on the days of data collection, the final sample consisted of 117 students (45.3% girls; mean age = 12 years, 3 months, *SD* = 9 months). All the students provided verbal assent to participate in this study.

Concerning socio-economic background, measured through the Family Affluence Scale (FAS; [37]), the majority of the participants came from medium- and high-class families (low FAS: 3.5%; medium FAS: 58.2%; high FAS: 38.3%). Consistent with national statistics about student population [38], the 81.2% of the participants had both parents born in Italy, 13.7% of them had one parent born outside Italy, and 5.1% had both parents born in foreign countries.

### Measures and procedure

#### Behavior during bullying episodes

Participants were presented with sixteen behavioral descriptions (adapted from [39–40]) and asked to nominate an unlimited number of classmates who better fitted each of them. Specifically, four items for each behavior (i.e., bullying, victimization, defending, and passive bystanding) were used (see the S1 Appendix for the complete list of items). In order to assure anonymity, students nominated classmates by indicating their corresponding number of the class roster. For each behavior a continuous score was computed by dividing the mean number of nominations received in the four items of each scale by the number of nominators. All scores showed satisfactory levels of internal consistency reliability (i.e, Cronbach’s alphas were .92, .85, .91, .68 and McDonald’s omegas were .93, .88, .92, .70 for bullying, victimization, defending, and passive bystanding, respectively).

#### Emotion Recognition Task

To assess the ability to recognize and label facial emotional expressions, the Emotion Recognition Task (ERT) was individually administered in a quiet room in the school. The ERT is a computerized paradigm in which morphed video clips of the six basic facial emotional expressions are presented at different intensities (40, 60, 80 and 100%; [21, 23]) by four actors, for a total of 96 trials. Each clip shows a face gradually changing from a neutral expression to one of the six emotions at a different level of intensity. Participants are asked to label each expression using a six-alternative force choice response without time restriction. The order of presentation of the morphs was fixed for all participants, starting with the lower intensities. Instructions and other technical aspects (e.g., number of frames, length of the videos) are detailed in Kessels’ and colleagues’ [23] paper. For sake of simplicity and parsimony, and based on preliminary analyses indicating that intensity had a substantially dichotomous effect on emotion recognition, in the following analyses intensity was split into low (40–60%) and high (80–100%) intensity.

### Statistical approach

Given the complex structure of our data, a Bayesian Generalized Mixed-Effects Models approach was used. Specifically, data were characterized by the presence of: (1) a dichotomous dependent variable (i.e., accuracy); (2) observations nested within subjects; (3) between- and within-subjects factors; (4) quantitative independent variables. Furthermore, to evaluate our research questions we needed to test and explore 2-way, 3-way and 4-way interactions between independent variables, and to compare several models.

As well documented in the statistical literature (e.g., [41–42]; see [43–44] for recent applications in psychology), the Bayesian approach is a valid alternative to the traditional frequentist approach to deal with our data structure and research questions. Without going into philosophical reasons, which are beyond the scope of the present paper, the Bayesian approach allows to: (1) accurately estimate mixed-effects models as suggested by Bolker and colleagues [45]; (2) coherently assess the variability of parameter estimates and provide associated inference via 95% Bayesian Credible Intervals (BCI). BCIs provide a direct representation of the most credible values of the estimated parameters given the prior distribution of the parameters and the observed data incorporated into the model. As a result, BCIs permit probabilistic statements to be made regarding confidence that the estimated parameters fall within any particular range. This is similar to the way researchers often misinterpret frequentist confidence intervals [44]. Therefore, Bayesian modeling allows us to interpret results in a manner that is both intuitive and more rational than common alternatives (see also [43]). BCIs were calculated using the percentile method; (3) compare the models in terms of evidence within a unified framework. In particular, the Watanabe-Akaike Information Criterion (WAIC) was used to select the best model among a set of candidate models fitted to the same data, and WAIC-weights are presented to compare the evidence of each model with regard to all candidate models. With this method, models were compared using a continuous and informative measure (i.e., evidence), rather than a series of simplified accept-reject dichotomous decisions typically adopted with the Null Hypothesis Significance Testing approach [42]; (4) appropriately evaluate the interaction effects using posterior distributions of planned comparisons between estimated parameters [46].

Two sets of analyses were conducted. First, we focused on performances in the Emotion Recognition Task, considering type and intensity of emotion, and controlling for participants’ gender. This allowed both to investigate our first goal (i.e., to replicate findings concerning differences in recognition accuracy depending on specific emotions), and to verify for the first time the accuracy pattern in a sample of Italian young adolescents. Three hypothesized logistic mixed-effects models were estimated and compared. The most plausible model was interpreted by means of estimated parameters, graphical representations and planned comparisons. Second, in order to answer our main research questions, participants’ behaviors during bullying episodes (i.e., bullying, victimization, defending, and passive bystanding) were evaluated as predictors of accuracy. As a measure of effect size, Odds Ratios and associated 95% BCIs are presented and discussed.

We estimated our models using the no-U-turn sampler [47], a variant of Hamiltonian Monte Carlo [48] as implemented in the STAN probabilistic programming language [49]. The basic idea is to iteratively poll possible parameter values from pre-specified prior distributions until convergence upon those model parameters that optimally represent the data.

We used the default prior specifications of the *R* package *brms* [50–51]. These priors could be considered less informative, and lead to posterior distributions of estimated parameters that are mostly influenced by the observed data rather than by prior information outside the study of interest. In our case, as stated below, this choice allowed to have appropriate parameter estimates and yielded satisfactory convergence of all tested models. Furthermore, from a reproducibility perspective, default priors allow other researchers to immediately reproduce our analyses and results.

Iterations of the estimation procedure were, as usual, split among independent “chains”. The purpose of including independent chains is to ensure that the model reliably converges on the same parameters. However, because each chain is initialized with random starting parameters, they require a certain number of iterations before the optimal solution is reached—after which the posterior distribution is sampled directly and used for inference purposes. To ensure exclusion of this “warm-up” (also known as “burn-in”) period, we discarded the initial samples from each chain prior to collapsing the chains for analysis [43]. All our models included 4 chains of 2,000 iterations each (8,000 in total) with a “warm-up” period of 1,000 iterations per chain (4,000 total) resulting in 4,000 usable samples.

Convergence was evaluated via visual inspection of the chains and using the Gelman-Rubin convergence statistic, R-hat, with values around 1 indicating convergence, and 1.100 considered as acceptable limit [41]. According to these diagnostics our models showed satisfactory convergence, with stationary distributions of estimated parameters and all associated R-hat’s ≤ 1.017. All related graphics and indices are available upon request from the authors.

Moreover, all models were also estimated with the traditional maximum likelihood approach using the lme4 package of R. In several cases, convergence was not reached. Overall, estimated model parameters were very similar to those produced by the Bayesian approach. Results of these analyses are available from the authors upon request

## Results

### Performances in the Emotion Recognition Task

Overall, the mean proportion of accuracy in emotion recognition was .62 (*SD* = .30). As for intensity of emotion, the marginal mean accuracy was .56 (*SD* = .29) for low intensity and .66 (*SD* = .30) for high intensity. For types of emotion, the marginal mean accuracy was .74 (*SD* = .23) for anger, .68 (*SD* = .27) for disgust, .39 (*SD* = .27) for fear, .44 (*SD* = .27) for sadness, .91 (*SD* = .13) for happiness, and .52 (*SD* = .22) for surprise. Overall, girls showed higher mean accuracy (.70, *SD* = .28) than boys (.56, *SD* = .30). In Table 1, the mean proportion of accuracy in emotion recognition by intensity, type of emotion and gender is presented.

**Table 1:** Mean proportion of accuracy in emotion recognition by intensity, emotion and gender.

Gender	Intensity	Emotion					
		Anger	Disgust	Fear	Sadness	Happiness	Surprise
Boys	Low	.58	51	.31	.31	.84	.49
	High	.79	.75	.32	.43	.96	.48
Girls	Low	.74	.68	.47	.47	.88	.55
	High	.86	.79	.50	.56	.97	.77

*n*_*subjects*_ = 117

Three plausible Bayesian logistic mixed-effects models were performed to analyze the data. In each model, the dependent variable was accuracy in emotion recognition (0 = incorrect, 1 = correct). The first baseline-reference model (M1) was a null model including only the random effect of subjects (i.e., a random intercept term for subjects was used). In the second model (M2), intensity and type of emotion as well as gender were added as main fixed effects. Finally, in the third model (M3) the interaction between intensity and type of emotion was also added. Results indicated that M3 (see Table 2) was clearly the most plausible model that has generated the observed data, having the lower WAIC (*WAIC*_*M1*_ = 14,565, *WAIC*_*M2*_ = 12,600, *WAIC*_*M3*_ = 12,499) and a probability of being the best of .99.

**Table 2:** Estimated parameters of the best fitting Bayesian logistic mixed-effects model with accuracy in emotion recognition as dependent variable (M3).

Fixed effects				
	*B*	*SE*	95% BCI	Odds Ratio (95% BCI)
Gender (girl)	.57	.10	.37 –.77	1.77 (1.45–2.16)
Intensity (high)	.96	.11	.75–1.18	2.62 (2.11–3.25)
Emotion				
Disgust	-.31	.10	-.50 –-.11	.74 (.61 –.90)
Fear	-1.20	.10	-1.39 –-1.01	.30 (.25 –.37)
Happiness	1.25	.12	1.02–1.48	3.48 (2.78–4.38)
Sadness	-1.17	.10	-1.36 –-.98	.31 (.26 –.38)
Surprise	-.61	.10	-.79 –-.42	.55 (.45 –.66)
Intensity × Emotion				
High × Disgust	-.02	.15	-.33 –.27	.98 (.72–1.31)
High × Fear	-.88	.15	-1.16 –-.59	.42 (.31 –.55)
High × Happiness	.65	.23	.21–1.10	1.92 (1.24–3.01)
High × Sadness	-.52	.15	-.81 –-.22	.60 (.45 –.80)
High × Surprise	-.94	.14	-1.22 –-0.66	.39 (.29 –.52)
Random effects				
	Estimate	*SE*	95% BCI	
${\hat{\sigma }}_{subjects}$	.50	.04	.43 –.59	

n_subjects_ = 117; n_observations_ = 11232

Baseline category for Gender was “boy”. Baseline category for Intensity was “low”. Baseline category for Emotion was “Anger”. BCI = Bayesian Credible Intervals

**Fig 1:**
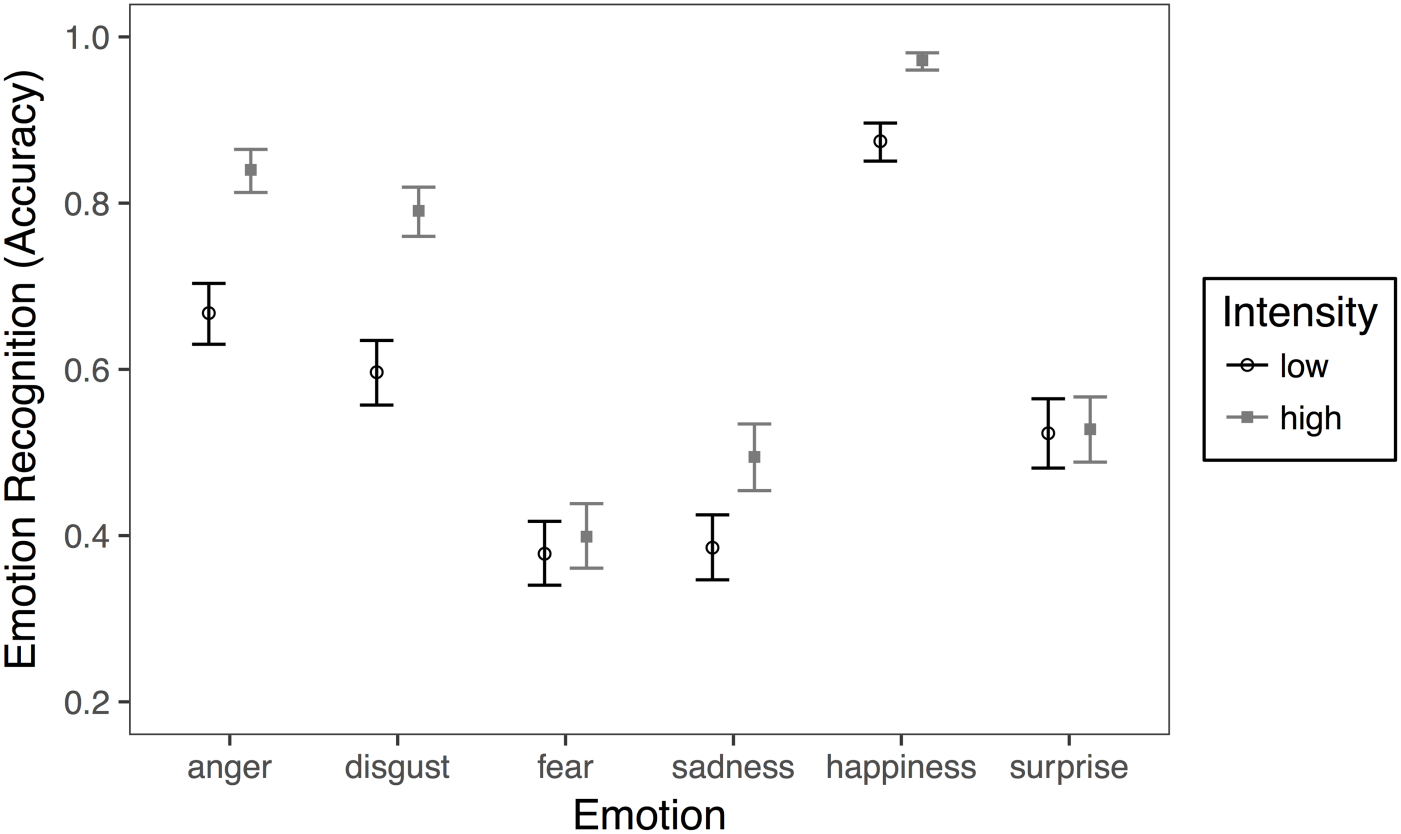
Estimated mean proportions of correct responses by emotion and intensity. Error bars represent 95% Bayesian credible intervals (n_subjects_ = 117, n_observations_ = 11232).

Beyond the effect of gender (*OR*_*girls vs boys*_ = 1.774; *95%BCI* = 1.45–2.16), the recognition of emotion was moderated by intensity. The interaction effect between intensity and type of emotion is depicted in Fig 1.

Bayesian comparisons across intensity showed that anger (95%*BCI* = .134–.211), disgust (95%*BCI* = .153–.236), happiness (95%*BCI* = .075–.121), and sadness (95%*BCI* = .065–.156) were better recognized in the high intensity condition than in the low intensity condition. No differences were found for fear (95%*BCI* = -.024–.066) and surprise (95%*BCI* = -.042–.051). Bayesian pairwise comparisons across emotions by type of intensity are presented in Table 3, for the interested readers.

**Table 3:** Bayesian pairwise comparisons across emotions by type of intensity with accuracy in emotion recognition as dependent variable.

Intensity	Pair of Emotions	Value	95% BCI
Low	anger—disgust	.07	.*03*–.*12*
	anger—fear	.29	.*25*–.*33*
	anger—happiness	-.21	-.*25*–-.*17*
	anger—sadness	.28	.*24*–.*33*
	anger—surprise	.14	.*10*–.*19*
	disgust—fear	.22	.*17*–.*26*
	disgust—happiness	-.28	-.*32–-*.*24*
	disgust—sadness	.21	.*16*–.*26*
	disgust—surprise	.07	.*03*–.*12*
	fear—happiness	-.50	-.*54–-*.*46*
	fear—sadness	-.01	-.05–.04
	fear—surprise	-.14	-.*19–-*.*10*
	happiness—sadness	.49	.*45*–.*53*
	happiness—surprise	.35	.*31*–.*39*
	sadness—surprise	-.14	-.*18–-*.*09*
High	anger—disgust	.05	.*01*–.*08*
	anger—fear	.44	.*40*–.*48*
	anger—happiness	-.13	-.*16–-*.*11*
	anger—sadness	.35	.*30*–.*39*
	anger—surprise	.31	.*27*–.*35*
	disgust—fear	.39	.*35*–.*43*
	disgust—happiness	-.18	-.*21–-*.*15*
	disgust—sadness	.3	.*25*–.*34*
	disgust—surprise	.26	.*22*–.*31*
	fear—happiness	-.57	-.*61–-*.*53*
	fear—sadness	-.10	-.*14–-*.*05*
	fear—surprise	-.13	-.*17–-*.*08*
	happiness—sadness	.48	.*44*–.*52*
	happiness—surprise	.44	.*41*–.*48*
	sadness—surprise	-.03	-.08–.01

n_subjects_ = 117; n_observations_ = 11232

Values are reported on the logit scale. For each pair of emotion, a positive value indicates that first emotion was recognized more accurately than the second emotion. BCI = Bayesian Credible Intervals. 95% BCIs that did not included 0 are reported in italics.

### Relations between emotion recognition and behaviors during bullying episodes

For the sake of transparency, in Table 4 descriptive statistics of emotion recognition accuracy by levels of behaviors, type and intensity of emotion, and participants’ gender, are shown.

**Table 4:** Mean proportion of accuracy in emotion recognition by intensity, emotion, gender and behaviors during bullying episodes (i.e., bullying/ /victimization/ defending/passive bystanding score). Medians were calculated separately for boys and girls.

***Bullying***								
Gender	Intensity	Bullying Score	Emotion					
			Anger	Disgust	Fear	Sadness	Happiness	Surprise
Boys	Low	Below median	.56	.53	.24	.31	.85	.46
		Above median	.60	.48	.38	.32	.84	.51
	High	Below median	.82	.73	.22	.44	.95	.44
		Above median	.77	.78	.42	.42	.98	.51
Girls	Low	Below median	.75	.76	.49	.55	.86	.56
		Above median	.72	.59	.45	.40	.90	.54
	High	Below median	.83	.83	.49	.62	.97	.57
		Above median	.89	.75	.50	.50	.97	.57
***Victimization***								
Gender	Intensity	Victimization Score	Emotion					
			Anger	Disgust	Fear	Sadness	Happiness	Surprise
Boys	Low	Below median	.61	.47	.35	.34	.86	.48
		Above median	.55	.54	.27	.29	.83	.49
	High	Below median	.79	.70	.32	.48	.95	.54
		Above median	.80	.80	.32	.38	.98	.42
Girls	Low	Below median	.78	.75	.51	.56	.87	.56
		Above median	.69	.61	.43	.38	.89	.54
	High	Below median	.88	.82	.55	.67	.99	.64
		Above median	.85	.76	.44	.44	.96	.50
***Defending***								
Gender	Intensity	Defending Score	Emotion					
			Anger	Disgust	Fear	Sadness	Happiness	Surprise
Boys	Low	Below median	.53	.48	.25	.34	.84	.49
		Above median	.63	.53	.37	.29	.85	.48
	High	Below median	.80	.76	.28	.49	.98	.50
		Above median	.78	.74	.36	.37	.94	.45
Girls	Low	Below median	.63	.57	.36	.34	.89	.51
		Above median	.86	.80	.59	.62	.86	.60
	High	Below median	.86	.76	.40	.43	.95	.50
		Above median	.86	.82	.60	.70	1.00	.66
***Passive bystanding***								
Gender	Intensity	Passive Bystanding Score	Emotion					
			Anger	Disgust	Fear	Sadness	Happiness	Surprise
Boys	Low	Below median	.59	.52	.31	.28	.85	.47
		Above median	.57	.49	.30	.35	.84	.50
	High	Below median	.79	.73	.29	.39	.94	.44
		Above median	.79	.78	.36	.47	.98	.52
Girls	Low	Below median	.77	.69	.52	.52	.89	.52
		Above median	.70	.66	.41	.43	.87	.58
	High	Below median	.88	.75	.53	.59	.97	.56
		Above median	.85	.84	.46	.52	.97	.58

n_subjects_ = 117

The division in low/high behavioral level (on the basis of the median) was made to facilitate interpretability and to provide the reader with a first picture of the relations among variables.

To examine the associations between emotion recognition and behaviors during bullying episodes, we started by comparing two Bayesian logistic mixed-effects models for each behavior with emotion recognition as dependent variable. The first model included participants’ gender, intensity and type of emotion, the participant score on the behavior of interest and the related 2-, 3- and 4-way interactions as fixed effects. Additionally, the scores on the other three behaviors were included (and thus controlled for) as main fixed effects. This approach allowed to partially overcome issues regarding model complexity (in terms of number of parameters), multicollinearity among behaviors (correlations are reported in Table 5), and interpretability of results. In the second model, the 4-way interaction was dropped. In both models, subjects were treated as random effects (i.e., a random intercept term for subjects was used).

**Table 5:** Means, standard deviations, and correlations between behaviors during bullying episodes scores.

	*M*	*SD*	1	2	3
1. Bullying	.12	.15			
2. Victimization	.11	.14	.02		
3. Defending	.18	.14	-.33**	-.28**	
4. Passive Bystanding	.21	.08	.40**	.04	-.49**

** indicates *p* < .01 (*n* = 117)

According to common guidelines [42, 52], model comparisons showed that (i) for bullying and defending, the model with the 4-way interaction should be strongly preferred; (ii) for victimization, the model with the 4-way interaction and the one without it were substantially equally plausible; (iii) for passive bystanding, there was weak evidence in favor of the model without the 4-way interaction (see Table 6).

**Table 6:** Comparison of Bayesian logistic mixed-effects models with emotion recognition as dependent variable by behavior during bullying episodes.

Behavior	Model	*WAIC*	Evidence Ratio in favor of the model including the 4-way interaction
Bullying	With the 4-way interaction	12,395.14	1,353.34
	Without the 4-way interaction	12,410.56	
Victimization	With the 4-way interaction	12,470.96	1.52
	Without the 4-way interaction	12,471.80	
Defending	With the 4-way interaction	12,456.48	21.09
	Without the 4-way interaction	12,462.58	
Passive Bystanding	With the 4-way interaction	12,503.01	.26
	Without the 4-way interaction	12,500.30	

n_subjects_ = 117, n_observations_ = 11232

*WAIC* = Watanabe-Akaike Information Criterion.

Consistent with these results and to facilitate interpretation, we chose to focus on the models that included the 4-way interactions (see Table 7).

**Table 7:** Estimated parameters of the Bayesian logistic mixed-effects models including four-way interactions (gender × intensity × emotion × behavior) with accuracy in emotion recognition as dependent variable.

***Bullying***				
Fixed effects				
	*B*	*SE*	95% BCI	Odds Ratio (95% BCI)
Gender (girl)	.55	.23	.08–1	1.73 (1.09–2.72)
Intensity (high)	1.23	.20	.85–1.61	3.43 (2.33–5.02)
Emotion				
Disgust	-.01	.17	-.34 –.33	.99 (.71–1.39)
Fear	-1.69	.19	-2.06 –-1.33	.18 (.13 –.26)
Happiness	1.59	.21	1.18–1.99	4.91 (3.27–7.32)
Sadness	-1.14	.18	-1.49 –-.78	.32 (.23 –.46)
Surprise	-.37	.17	-.71 –-.03	.69 (.49 –.97)
Bullying	.22	.62	-.95–1.45	1.02 (.91–1.16)
Victimization	2.25	.49	1.24–3.21	1.25 (1.13–1.38)
Defending	-.80	.35	-1.46 –-.1	.92 (.86 –.99)
Passive Bystanding	.84	.73	-.57–2.3	1.09 (.95–1.26)
Gender × Intensity	-.99	.31	-1.6 –-.39	.37 (.2 –.67)
Gender × Emotion				
Girls × Disgust	-.11	.27	-.66 –.41	.9 (.52–1.51)
Girls × Fear	.40	.27	-.15 –.91	1.49 (.86–2.48)
Girls × Happiness	-1.09	.32	-1.71 –-.44	.34 (.18 –.64)
Girls × Sadness	.06	.27	-.48 –.6	1.07 (.62–1.82)
Girls × Surprise	-.66	.26	-1.19 –-.13	.52 (.3 –.88)
Gender × Bullying	-1.42	1.59	-4.49–1.69	.87 (.64–1.18)
Intensity × Emotion				
High × Disgust	-.52	.27	-1.06 –.03	.6 (.35–1.03)
High × Fear	-1.17	.28	-1.71 –-.63	.31 (.18 –.53)
High × Happiness	-.19	.41	-.98 –.62	.83 (.37–1.86)
High × Sadness	-.66	.27	-1.2 –-.14	.52 (.3 –.87)
High × Surprise	-1.34	.26	-1.85 –-.85	.26 (.16 –.43)
Intensity × Bullying	-1.14	.79	-2.67 –.41	.89 (.77–1.04)
Emotion × Bullying				
Disgust × Bullying	-1.97	.74	-3.46 –-.56	.82 (.71 –.95)
Fear × Bullying	2.94	.74	1.5–4.4	1.34 (1.16–1.55)
Happiness × Bullying	-1.08	.83	-2.69 –.56	.9 (.76–1.06)
Sadness × Bullying	-.09	.75	-1.59–1.39	.99 (.85–1.15)
Surprise × Bullying	-.16	.72	-1.54–1.27	.98 (.86–1.14)
Gender × Intensity × Emotion				
Girls × High × Disgust	.62	.42	-.21–1.45	1.86 (.81–4.26)
Girls × High × Fear	.96	.41	.17–1.76	2.62 (1.19–5.79)
Girls × High × Happiness	2.00	.64	.74–3.29	7.38 (2.1–26.93)
Girls × High × Sadness	.79	.40	.01–1.61	2.21 (1.01–4.98)
Girls × High × Surprise	1.14	.39	.37–1.92	3.14 (1.45–6.81)
Gender × Intensity × Bullying	12.39	3.11	6.55–18.64	3.45 (1.93–6.45)
Gender × Emotion × Bullying				
Girls × Disgust × Bullying	-.61	1.94	-4.46–3.27	.94 (.64–1.39)
Girls × Fear × Bullying	-2.25	1.89	-5.74–1.59	.8 (.56–1.17)
Girls × Happiness × Bullying	9.91	3.11	4.17–16.31	2.69 (1.52–5.11)
Girls × Sadness × Bullying	-2.41	2.00	-6.27–1.54	.79 (.53–1.17)
Girls × Surprise × Bullying	2.16	1.89	-1.66–5.93	1.24 (.85–1.81)
Intensity × Emotion × Bullying				
High × Disgust × Bullying	3.96	1.17	1.72–6.23	1.49 (1.19–1.86)
High × Fear × Bullying	1.19	1.10	-.89–3.32	1.13 (.91–1.39)
High × Happiness × Bullying	6.05	2.53	1.59–11.67	1.83 (1.17–3.21)
High × Sadness × Bullying	.81	1.09	-1.36–2.91	1.08 (.87–1.34)
High × Surprise × Bullying	1.57	1.06	-.51–3.65	1.17 (.95–1.44)
Gender × Intensity × Emotion × Bullying				
Girls × High × Disgust × Bullying	-11.06	3.76	-18.82 –-3.54	.33 (.15 –.7)
Girls × High × Fear × Bullying	-11.26	3.57	-18.29 –-4.52	.32 (.16 –.64)
Girls × High × Happiness × Bullying	-24.33	5.95	-36.13 –-12.61	.09 (.03 –.28)
Girls × High × Sadness × Bullying	-11.93	3.68	-19.31 –-4.79	.3 (.15 –.62)
Girls × High × Surprise × Bullying	-12.17	3.58	-19.37 –-5.39	.3 (.14 –.58)
*Random effects*				
	Estimate	SE	*95% BCI*	
${\hat{\sigma }}_{subjects}$	.43	.04	.36 –.51	
***Victimization***				
Fixed effects				
	*B*	*SE*	95% BCI	Odds Ratio (95% BCI)
Gender (girl)	.52	.22	.08 –.95	1.68 (1.08–2.58)
Intensity (high)	1.05	.19	.66–1.42	2.87 (1.94–4.14)
Emotion				
Disgust	-.58	.17	-.91 –-.26	.56 (.4 –.77)
Fear	-1.04	.17	-1.38 –-.71	.35 (.25 –.49)
Happiness	1.43	.20	1.04–1.83	4.18 (2.82–6.24)
Sadness	-1.20	.18	-1.55 –-.85	.3 (.21 –.43)
Surprise	-.64	.16	-.96 –-.32	.53 (.38 –.73)
Victimization	-.83	.70	-2.25 –.53	.92 (.8–1.05)
Defending	2.09	.46	1.19–3.03	1.23 (1.13–1.35)
Bullying	.42	.33	-.21–1.04	1.04 (.98–1.11)
Passive Bystanding	.78	.68	-.52–2.1	1.08 (.95–1.23)
Gender × Intensity	-.12	.31	-.72 –.48	.89 (.49–1.62)
Gender × Emotion				
Girls × Disgust	.40	.26	-.1 –.94	1.5 (.9–2.55)
Girls × Fear	-.23	.26	-.72 –.3	.8 (.49–1.35)
Girls × Happiness	-.89	.31	-1.5 –-.28	.41 (.22 –.75)
Girls × Sadness	.19	.27	-.34 –.69	1.2 (.72–1.99)
Girls × Surprise	-.26	.25	-.76 –.21	.77 (.47–1.23)
Gender × Victimization	-.46	1.34	-3.05–2.18	.96 (.74–1.24)
Intensity × Emotion				
High × Disgust	.07	.26	-.44 –.59	1.07 (.64–1.81)
High × Fear	-1.25	.25	-1.74 –-.75	.29 (.18 –.47)
High × Happiness	.24	.39	-.51–1.04	1.27 (.6–2.84)
High × Sadness	-.38	.26	-.89 –.15	.68 (.41–1.16)
High × Surprise	-.81	.25	-1.28 –-.32	.44 (.28 –.73)
Intensity × Victimization	-.02	.94	-1.81–1.84	1 (.83–1.2)
Emotion × Victimization				
Disgust × Victimization	2.12	.86	.51–3.86	1.24 (1.05–1.47)
Fear × Victimization	-1.39	1.01	-3.44 –.55	.87 (.71–1.06)
Happiness × Victimization	-.07	.99	-1.94–1.92	.99 (.82–1.21)
Sadness × Victimization	.33	.94	-1.54–2.15	1.03 (.86–1.24)
Surprise × Victimization	1.90	.86	.23–3.59	1.21 (1.02–1.43)
Gender × Intensity × Emotion				
Girls × High × Disgust	-.35	.43	-1.21 –.5	.7 (.3–1.65)
Girls × High × Fear	.46	.40	-.32–1.24	1.58 (.73–3.44)
Girls × High × Happiness	1.37	.69	.04–2.74	3.93 (1.04–15.45)
Girls × High × Sadness	-.11	.41	-.92 –.66	.89 (.4–1.93)
Girls × High × Surprise	.20	.40	-.59 –.97	1.22 (.55–2.64)
Gender × Intensity × Victimization	-.85	1.81	-4.31–2.73	.92 (.65–1.31)
Gender × Emotion × Victimization				
Girls × Disgust × Victimization	-3.53	1.63	-6.73 –-.3	.7 (.51 –.97)
Girls × Fear × Victimization	1.78	1.71	-1.65–5.02	1.2 (.85–1.65)
Girls × Happiness × Victimization	6.17	2.41	1.68–11.01	1.85 (1.18–3.01)
Girls × Sadness × Victimization	-3.04	1.74	-6.57 –.27	.74 (.52–1.03)
Girls × Surprise × Victimization	-1.77	1.60	-4.87–1.29	.84 (.61–1.14)
Intensity × Emotion × Victimization				
High × Disgust × Victimization	.24	1.37	-2.48–2.91	1.02 (.78–1.34)
High × Fear × Victimization	2.30	1.39	-.45–5.04	1.26 (.96–1.66)
High × Happiness × Victimization	2.83	2.31	-1.4–7.76	1.33 (.87–2.17)
High × Sadness × Victimization	-1.24	1.37	-3.9–1.36	.88 (.68–1.15)
High × Surprise × Victimization	-2.19	1.27	-4.61 –.23	.8 (.63–1.02)
Gender × Intensity × Emotion × Victimization				
Girls × High × Disgust × Victimization	.66	2.52	-4.13–5.61	1.07 (.66–1.75)
Girls × High × Fear × Victimization	-1.77	2.47	-6.48–3.14	.84 (.52–1.37)
Girls × High × Happiness × Victimization	-11.25	4.10	-19.27 –-3.34	.32 (.15 –.72)
Girls × High × Sadness × Victimization	1.43	2.56	-3.42–6.4	1.15 (.71–1.9)
Girls × High × Surprise × Victimization	.39	2.38	-4.32–5.1	1.04 (.65–1.67)
*Random effects*				
	Estimate	SE	*95% BCI*	
${\hat{\sigma }}_{subjects}$	.41	.04	.34 –.49	
***Defending***				
Fixed effects				
	*B*	*SE*	95% BCI	Odds Ratio (95% BCI)
Gender (girl)	-.08	.33	-.74 –.56	.92 (.48–1.75)
Intensity (high)	1.32	.28	.77–1.84	3.75 (2.16–6.32)
Emotion				
Disgust	-.11	.25	-.61 –.36	.89 (.55–1.44)
Fear	-1.30	.26	-1.81 –-.8	.27 (.16 –.45)
Happiness	1.66	.29	1.09–2.22	5.27 (2.97–9.18)
Sadness	-.76	.26	-1.28 –-.25	.47 (.28 –.78)
Surprise	-.11	.25	-.6 –.37	.89 (.55–1.44)
Defending	1.75	1.60	-1.3–4.93	1.19 (.88–1.64)
Bullying	.28	.32	-.37 –.9	1.03 (.96–1.09)
Victimization	-.85	.34	-1.5 –-.16	.92 (.86 –.98)
Passive Bystanding	.51	.71	-.9–1.89	1.05 (.91–1.21)
Gender × Intensity	.73	.46	-.18–1.62	2.07 (.84–5.06)
Gender × Emotion				
Girls × Disgust	-.19	.41	-.99 –.61	.83 (.37–1.83)
Girls × Fear	.29	.41	-.51–1.12	1.34 (.6–3.06)
Girls × Happiness	.50	.47	-.44–1.45	1.64 (.65–4.24)
Girls × Sadness	-.77	.43	-1.59 –.09	.47 (.2–1.09)
Girls × Surprise	.08	.40	-.67 –.88	1.09 (.51–2.4)
Gender × Defending	2.46	1.88	-1.1–6.14	1.28 (.9–1.85)
Intensity × Emotion				
High × Disgust	-.26	.39	-1 –.53	.77 (.37–1.7)
High × Fear	-1.11	.39	-1.85 –-.35	.33 (.16 –.71)
High × Happiness	1.46	.58	.35–2.6	4.3 (1.42–13.44)
High × Sadness	-.81	.39	-1.58 –-.05	.44 (.21 –.95)
High × Surprise	-1.47	.37	-2.19 –-.74	.23 (.11 –.48)
Intensity × Defending	-2.41	2.07	-6.32–1.73	.79 (.53–1.19)
Emotion × Defending				
Disgust × Defending	-1.79	1.88	-5.44–1.9	.84 (.58–1.21)
Fear × Defending	.93	1.93	-2.84–4.84	1.1 (.75–1.62)
Happiness × Defending	-2.15	2.16	-6.24–2.3	.81 (.54–1.26)
Sadness × Defending	-3.52	2.02	-7.46 –.46	.7 (.47–1.05)
Surprise × Defending	-2.47	1.89	-6.13–1.25	.78 (.54–1.13)
Gender × Intensity × Emotion				
Girls × High × Disgust	-.66	.63	-1.88 –.62	.52 (.15–1.85)
Girls × High × Fear	-.76	.62	-1.95 –.47	.47 (.14–1.6)
Girls × High × Happiness	-3.37	.90	-5.15 –-1.58	.03 (.01 –.21)
Girls × High × Sadness	-.89	.64	-2.12 –.34	.41 (.12–1.4)
Girls × High × Surprise	-.88	.62	-2.09 –.34	.42 (.12–1.4)
Gender × Intensity × Defending	-2.54	2.46	-7.39–2.12	.78 (.48–1.24)
Gender × Emotion × Defending				
Girls × Disgust × Defending	1.74	2.26	-2.79–6.21	1.19 (.76–1.86)
Girls × Fear × Defending	-2.02	2.28	-6.71–2.39	.82 (.51–1.27)
Girls × Happiness × Defending	-2.65	2.51	-7.7–2.22	.77 (.46–1.25)
Girls × Sadness × Defending	4.44	2.37	-.25–9.02	1.56 (.98–2.46)
Girls × Surprise × Defending	-1.01	2.21	-5.35–3.23	.9 (.59–1.38)
Intensity × Emotion × Defending				
High × Disgust × Defending	3.12	2.94	-2.72–8.85	1.37 (.76–2.42)
High × Fear × Defending	1.15	2.87	-4.53–6.67	1.12 (.64–1.95)
High × Happiness × Defending	-5.82	3.55	-12.86–1.04	.56 (.28–1.11)
High × Sadness × Defending	2.47	2.92	-3.25–8.06	1.28 (.72–2.24)
High × Surprise × Defending	3.37	2.78	-2.12–8.76	1.4 (.81–2.4)
Gender × Intensity × Emotion × Defending				
Girls × High × Disgust × Defending	-.22	3.45	-7.06–6.43	.98 (.49–1.9)
Girls × High × Fear × Defending	3.56	3.31	-2.76–1.07	1.43 (.76–2.74)
Girls × High × Happiness × Defending	18.09	4.72	8.93–27.42	6.1 (2.44–15.52)
Girls × High × Sadness × Defending	2.69	3.41	-4.03–9.35	1.31 (.67–2.55)
Girls × High × Surprise × Defending	3.10	3.29	-3.28–9.59	1.36 (.72–2.61)
*Random effects*				
	Estimate	SE	*95% BCI*	
${\hat{\sigma }}_{subjects}$	.41	.04	.34 –.48	
***Passive bystanding***				
Fixed effects				
	*B*	*SE*	95% BCI	Odds Ratio (95% BCI)
Gender (girl)	.84	.52	-.18–1.85	2.32 (.84–6.38)
Intensity (high)	.89	.41	.08–1.68	2.43 (1.09–5.38)
Emotion				
Disgust	-.04	.37	-.75 –.7	.96 (.47–2.01)
Fear	-1.15	.39	-1.9 –-.38	.32 (.15 –.68)
Happiness	1.43	.42	.62–2.26	4.2 (1.86–9.61)
Sadness	-1.41	.38	-2.14 –-.66	.24 (.12 –.52)
Surprise	-.76	.37	-1.47 –-.05	.47 (.23 –.95)
Passive Bystanding	.42	1.27	-1.97–2.93	1.04 (.82–1.34)
Defending	2.24	.48	1.33–3.16	1.25 (1.14–1.37)
Bullying	.26	.34	-.43 –.92	1.03 (.96–1.1)
Victimization	-.83	.36	-1.54 –-.14	.92 (.86 –.99)
Gender × Intensity	-.77	.69	-2.11 –.61	.46 (.12–1.85)
Gender × Emotion				
Girls × Disgust	-.39	.62	-1.59 –.83	.68 (.2–2.28)
Girls × Fear	.11	.62	-1.11–1.3	1.12 (.33–3.65)
Girls × Happiness	-1.11	.72	-2.55 –.27	.33 (.08–1.31)
Girls × Sadness	.42	.63	-.84–1.66	1.53 (.43–5.24)
Girls × Surprise	-1.16	.61	-2.34 –.05	.31 (.1–1.06)
Gender × Passive Bystanding	-2.08	2.38	-6.77–2.6	.81 (.51–1.3)
Intensity × Emotion				
High × Disgust	-.30	.57	-1.39 –.83	.74 (.25–2.29)
High × Fear	-1.18	.58	-2.33 –-.05	.31 (.1 –.96)
High × Happiness	-.32	.83	-1.93–1.29	.73 (.14–3.65)
High × Sadness	-.55	.55	-1.62 –.56	.58 (.2–1.75)
High × Surprise	-1.24	.55	-2.34 –-.17	.29 (.1 –.84)
Intensity × Passive Bystanding	.71	1.64	-2.54–3.92	1.07 (.78–1.48)
Emotion × Passive Bystanding				
Disgust × Passive Bystanding	-1.20	1.50	-4.18–1.73	.89 (.66–1.19)
Fear × Passive Bystanding	-.14	1.57	-3.28–2.94	.99 (.72–1.34)
Happiness × Passive Bystanding	-.07	1.72	-3.39–3.28	.99 (.71–1.39)
Sadness × Passive Bystanding	1.15	1.55	-1.92–4.1	1.12 (.82–1.51)
Surprise × Passive Bystanding	1.59	1.47	-1.27–4.45	1.17 (.88–1.56)
Gender × Intensity × Emotion				
Girls × High × Disgust	-.44	.95	-2.34–1.43	.65 (.1–4.18)
Girls × High × Fear	1.10	.93	-.72–2.93	3.01 (.49–18.67)
Girls × High × Happiness	2.53	1.43	-.27–5.39	12.54 (.76–218.9)
Girls × High × Sadness	.40	.92	-1.42–2.15	1.5 (.24–8.59)
Girls × High × Surprise	1.81	.90	.03–3.56	6.13 (1.03–35.08)
Gender × Intensity × Passive Bystanding	3.06	3.22	-3.1–9.43	1.36 (.73–2.57)
Gender × Emotion × Passive Bystanding				
Girls × Disgust × Passive Bystanding	1.76	2.85	-3.81–7.35	1.19 (.68–2.08)
Girls × Fear × Passive Bystanding	-1.03	2.91	-6.65–4.67	.9 (.51–1.59)
Girls × Happiness × Passive Bystanding	3.55	3.38	-2.93–1.25	1.43 (.75–2.79)
Girls × Sadness × Passive Bystanding	-2.47	2.96	-8.27–3.43	.78 (.44–1.41)
Girls × Surprise × Passive Bystanding	3.81	2.83	-1.8–9.25	1.46 (.84–2.52)
Intensity × Emotion × Passive Bystanding				
High × Disgust × Passive Bystanding	1.70	2.31	-2.81–6.16	1.19 (.75–1.85)
High × Fear × Passive Bystanding	.82	2.34	-3.79–5.47	1.09 (.68–1.73)
High × Happiness × Passive Bystanding	4.22	3.66	-2.7–11.55	1.52 (.76–3.17)
High × Sadness × Passive Bystanding	.06	2.26	-4.46–4.54	1.01 (.64–1.57)
High × Surprise × Passive Bystanding	.66	2.21	-3.62–5.08	1.07 (.7–1.66)
Gender × Intensity × Emotion × Passive Bystanding				
Girls × High × Disgust × Passive Bystanding	1.21	4.45	-7.56–9.91	1.13 (.47–2.69)
Girls × High × Fear × Passive Bystanding	-4.22	4.33	-12.6–4.37	.66 (.28–1.55)
Girls × High × Happiness × Passive Bystanding	-11.38	6.64	-24.36–1.9	.32 (.09–1.21)
Girls × High × Sadness × Passive Bystanding	-1.72	4.36	-1.1–6.85	.84 (.36–1.98)
Girls × High × Surprise × Passive Bystanding	-7.61	4.18	-15.75 –.48	.47 (.21–1.05)
*Random effects*				
	Estimate	SE	*95% BCI*	
${\hat{\sigma }}_{subjects}$	.43	.04	.36 –.50	

n_subjects_ = 117, n_observations_ = 11232

Baseline category for Gender was “boy”. Baseline category for Intensity was “low”. Baseline category for Emotion was “Anger”. BCI = Bayesian Credible Intervals

For main and interaction effects including behavior (i.e., bullying, victimization, defending and passive bystanding), the Odds Ratio for a 10% increase in the associated score is presented.

**Fig 2:**
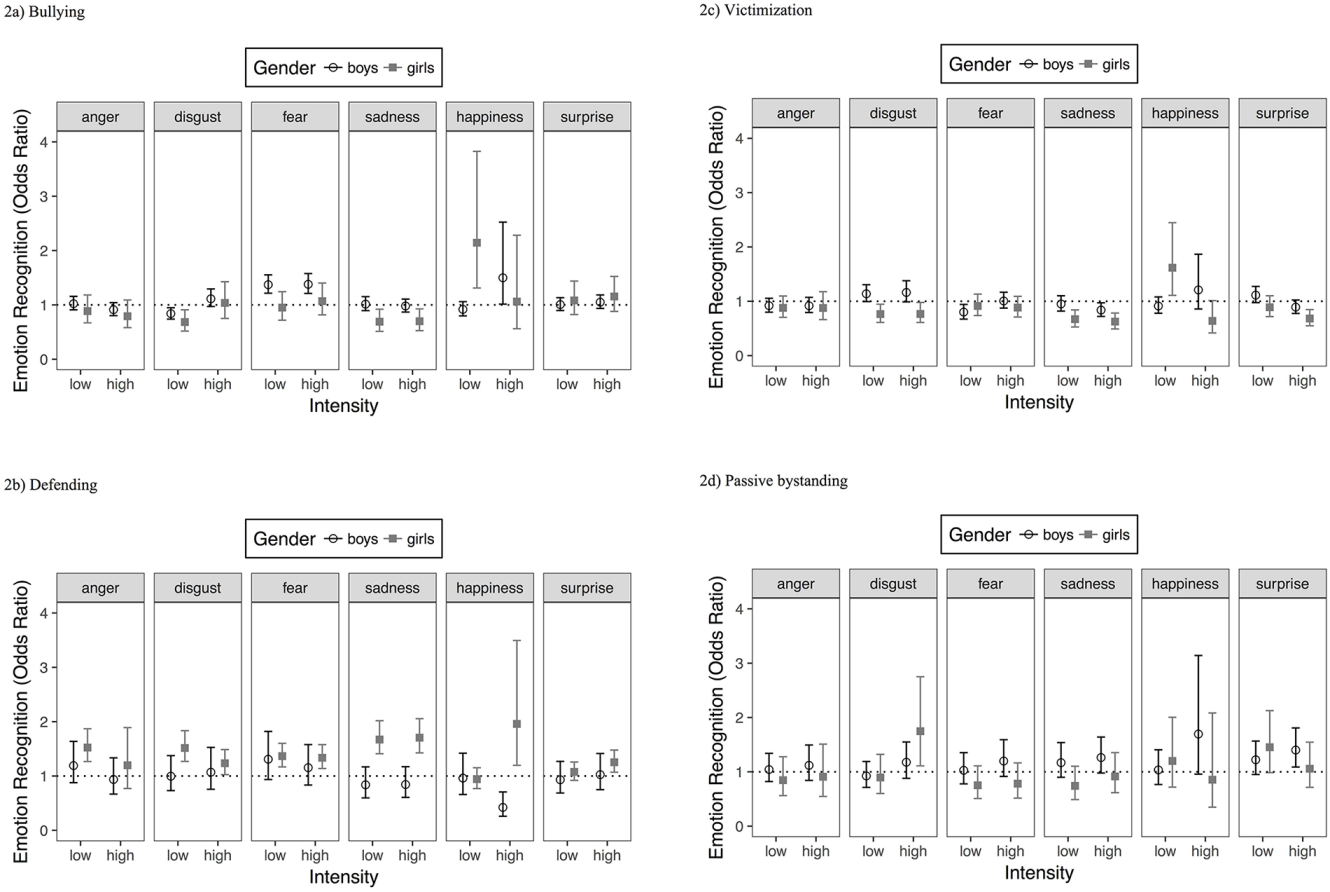
Estimated Odds Ratios representing the relative variation in the odds of emotion recognition accuracy for a 10% increase of bullying/ /victimization/ defending/passive bystanding score. Error bars represent 95% Bayesian credible intervals (n_subjects_ = 117, n_observations_ = 11232).

The estimated 4-way interactions for the four models are presented in Fig 2. For each combination of gender, intensity and type of emotion the Odds Ratio associated with an increment of 10% in the bullying (Fig 2a), victimization (Fig 2b), defending (Fig 2c), and passive bystanding (Fig 2d) score and the associated 95%BCI are displayed. All the corresponding numerical indices are included in Table 8.

**Table 8:** Estimated Odds Ratios for emotion recognition associated with an increase of 10% in behaviors during bullying episodes (i.e., bullying/ /victimization/ defending/passive bystanding score) by type of behavior, emotion, intensity and gender.

***Bullying***				
Emotion	Intensity	Gender	*Odds Ratio*	*95% BCI*
Anger	Low	Boys	1.02	.91–1.16
		Girls	.89	.67–1.18
	High	Boys	.91	.80–1.04
		Girls	.79	.58–1.09
Disgust	Low	Boys	.*84*	.*74 –*.*95*
		Girls	.*68*	.*52 –*.*91*
	High	Boys	1.11	.97–1.29
		Girls	1.04	.75–1.43
Fear	Low	Boys	*1*.*37*	*1*.*22–1*.*55*
		Girls	.95	.72–1.24
	High	Boys	*1*.*38*	*1*.*21–1*.*58*
		Girls	1.07	.82–1.40
Sadness	Low	Boys	1.01	.89–1.15
		Girls	.*69*	.*51 –*.*92*
	High	Boys	.98	.87–1.11
		Girls	.*70*	.*53 –*.*93*
Happiness	Low	Boys	.92	.80–1.06
		Girls	*2*.*14*	*1*.*31–3*.*83*
	High	Boys	*1*.*50*	*1*.*01–2*.*52*
		Girls	1.06	.56–2.28
Surprise	Low	Boys	1.01	.90–1.13
		Girls	1.08	.82–1.44
	High	Boys	1.05	.94–1.18
		Girls	1.15	.88–1.52
***Victimization***				
Emotion	Intensity	Gender	*Odds Ratio*	*95% BCI*
Anger	Low	Boys	.92	.80–1.05
		Girls	.88	.70–1.10
	High	Boys	.92	.79–1.07
		Girls	.88	.66–1.18
Disgust	Low	Boys	1.14	.99–1.31
		Girls	.*76*	.*61* –.*95*
	High	Boys	1.16	.99–1.38
		Girls	.*77*	.*61* –.*98*
Fear	Low	Boys	.*80*	.*67* –.*94*
		Girls	.91	.74–1.13
	High	Boys	1.01	.87–1.17
		Girls	.88	.71–1.09
Sadness	Low	Boys	.95	.82–1.10
		Girls	.*67*	.*53* –.*84*
	High	Boys	.*84*	.*72* –.*97*
		Girls	.*63*	.*49* –.*78*
Happiness	Low	Boys	.91	.78–1.08
		Girls	*1*.*62*	*1*.*11*–*2*.*45*
	High	Boys	1.21	.86–1.87
		Girls	.64	.42–1.01
Surprise	Low	Boys	1.11	.98–1.27
		Girls	.89	.72–1.10
	High	Boys	.89	.78–1.02
		Girls	.*68*	.*55* –.*85*
***Defending***				
Emotion	Intensity	Gender	*Odds Ratio*	*95% BCI*
Anger	Low	Boys	1.19	.88–1.64
		Girls	*1*.*52*	*1*.*27*–*1*.*87*
	High	Boys	.94	.67–1.33
		Girls	1.20	.77–1.89
Disgust	Low	Boys	1.00	.73–1.37
		Girls	*1*.*52*	*1*.*27*–*1*.*83*
	High	Boys	1.07	.76–1.53
		Girls	*1*.*23*	*1*.*02*–*1*.*49*
Fear	Low	Boys	1.31	.93–1.82
		Girls	*1*.*37*	*1*.*17*–*1*.*60*
	High	Boys	1.15	.83–1.58
		Girls	*1*.*34*	*1*.*14*–*1*.*58*
Sadness	Low	Boys	.84	.60–1.17
		Girls	*1*.*67*	*1*.*41*–*2*.*02*
	High	Boys	.84	.61–1.17
		Girls	*1*.*71*	*1*.*43*–*2*.*05*
Happiness	Low	Boys	.96	.66–1.42
		Girls	.94	.77–1.15
	High	Boys	.*42*	.*26* –.*71*
		Girls	*1*.*96*	*1*.*20*–*3*.*49*
Surprise	Low	Boys	.93	.69–1.27
		Girls	1.08	.92–1.26
	High	Boys	1.03	.75–1.41
		Girls	*1*.*25*	*1*.*07*–*1*.*48*
***Passive bystanding***				
Emotion	Intensity	Gender	*Odds Ratio*	*95% BCI*
Anger	Low	Boys	1.04	.82–1.34
		Girls	.85	.56–1.28
	High	Boys	1.12	.84–1.49
		Girls	.91	.55–1.51
Disgust	Low	Boys	.92	.71–1.19
		Girls	.90	.60–1.32
	High	Boys	1.18	.88–1.55
		Girls	*1*.*75*	*1*.*11*–*2*.*75*
Fear	Low	Boys	1.03	.78–1.35
		Girls	.75	.51–1.11
	High	Boys	1.20	.92–1.59
		Girls	.78	.52–1.16
Sadness	Low	Boys	1.17	.90–1.54
		Girls	.74	.49–1.10
	High	Boys	1.26	.98–1.64
		Girls	.92	.62–1.35
Happiness	Low	Boys	1.04	.77–1.41
		Girls	1.20	.72–2.00
	High	Boys	1.70	.96–3.14
		Girls	.86	.35–2.08
Surprise	Low	Boys	1.22	.95–1.57
		Girls	1.45	.98–2.13
	High	Boys	*1*.*40*	*1*.*09*–*1*.*81*
		Girls	1.06	.71–1.55

n_subjects_ = 117, n_observations_ = 11232

BCI = Bayesian Credible Intervals. 95% BCIs that did not included 1 are reported in italics.

#### Bullying

Ninety-five percent BCIs indicated that higher levels of bullying were associated with better recognition of fear in both intensity conditions among boys, with worse recognition of low and high intensity sadness among girls and with less accuracy in recognizing disgust in the low intensity condition in both gender groups. Moreover, bullying was associated to better recognition of happiness, at high intensity among boys and at low levels among girls (Table 7).

#### Victimization

Among boys, higher levels of victimization were associated with less accuracy in recognizing fear in the low intensity condition, and sadness in the high intensity condition. Among girls, higher levels of victimization were associated with less accuracy in recognizing disgust and sadness in both intensity conditions, surprise in the high intensity condition and with better recognition of happiness in the low intensity condition (Table 7).

#### Defending

Among girls, higher levels of defending were related to better recognition of anger in the low intensity condition, disgust, fear, and sadness in both intensity conditions and surprise at high intensity. Moreover, higher defending was associated with the recognition of happiness in the high intensity condition, so that it was lower among boys and higher among girls (Table 7).

#### Passive bystanding

Among girls, passive bystanding behavior was associated with more accuracy in recognizing disgust in the high intensity condition. Moreover, higher levels of passive bystanding in boys were related to better recognition of surprise in the high intensity condition (Table 7).

## Discussion

The aim of this study was to offer, for the first time, a fullest possible overview of the relation between facial emotion recognition abilities and young adolescents’ behavior during bullying episodes. In particular, four different behaviors in bullying (i.e., bullying others, being victimized, defending the victim, and passive bystanding) and recognition skills of morphed facial expressions of the six basic emotions, expressed at two different intensities, were considered. Given the complexity of both data structure and research questions, we used a Bayesian approach rather than the traditional frequentist approach. This represents an important novelty in the field. Beyond theoretical reasons, this approach allowed us to obtain robust estimates and model convergence in spite of the non-optimal sample size-number of estimated parameters ratio.

First, we verified that the accuracy in the facial emotion recognition, measured through the recently developed Emotion Recognition Task [21], paralleled previous findings in the literature. Results showed a similar pattern in the recognition of the six basic emotions, with higher mean accuracy in the recognition of happiness, anger and disgust, and lower performances concerning surprise, sadness and fear. Moreover, the importance of considering different emotion intensities [21, 23] was confirmed, in that, for four out of six emotions (i.e., anger, disgust, sadness, and happiness), intensity influenced the recognition performance. Finally, as expected, girls showed generally higher accuracy in recognizing emotions compared with boys (e.g., [36]).

Concerning the main goal of this study, which dealt with the relation between the recognition of negative facial emotions and young adolescents’ behaviors during bullying episodes, an interesting picture emerged. In general, one notable result concerned the recognition of emotions as an ability that can be related with both moral (i.e., defending the victim) and immoral (i.e., bullying others) behavior in the context of bullying dynamics. A prominent example is represented by fear recognition, which was positively related with both higher levels of bullying, among boys, and defending, among girls. We could hypothesize that recognizing fear may help aggressive youth to identify vulnerable victims and make the aggressive behavior more efficacious; at the same time, it could promote prosocial behavior, for example by alerting bystanders that something wrong (and potentially dangerous) is happening and eliciting their empathic responses towards victims.

A link with empathic skills could be also hypothesized analyzing sadness recognition in girls, which was negatively related to bullying and positively with defending. It could be speculated that detecting sadness in victims, even when not full-blown expressed, could elicit empathic concern for them and make clear that what is happening is neither pleasant nor desirable for the victims; this, in turn, could increase the likelihood of helping. Likewise, girls’ greater ability in recognizing disgust and low intensity anger could be positively associated with defending behavior because it allows to better understand hostile bullies’ intentions. Although we were not specifically interested in positive emotions, likely less crucial in bullying dynamics, it should be noticed that among girls defending was also associated with higher recognition of surprise and happiness. Thus, we may hypothesize that defending behavior in girls is connected with a general ability in recognizing facial emotions. Overall, these findings confirm the growing literature showing that defending behavior in bullying is more frequent among girls and is associated with a pattern of social-emotional skills [15, 29].

Conversely, our expectation on the associations between difficulties in recognizing negative emotions and higher levels of passive bystanding was not confirmed. Indeed, the only relevant result concerning passive bystanding behavior was a positive association with disgust recognition at high intensity among girls. This is another example of the idea that emotion recognition is a “neutral ability” that does not necessarily represent a driving force for moral and prosocial behavior.

Regarding victimization, consistent with Woods and colleagues’ study [12], our findings overall confirmed that higher levels of victimization were associated with a general difficulty in recognizing emotions. This result is not surprisingly and complements the large body of research that has documented the social-cognitive, emotional, and interpersonal deficiencies of frequently victimized youth (e.g., [26, 53–54]). However, the pattern of results with respect to specific emotions that emerged from our analysis was not clear-cut and easy to interpret; future studies should try to replicate the current findings and test more precise hypotheses about victims’ impairment in recognition of specific emotions, also adopting experimental (e.g., scenarios) and longitudinal designs.

This study has also some limitations. For example, the collected data were cross-sectional. Although from a theoretical point of view emotion recognition abilities can be more easily conceived as a precursor of a conduct rather than a consequence of behavior, the cross-sectional design of this study did not allow us to drive conclusions about the direction of the effects. Therefore, the model proposed in the present study will need to be retested with longitudinal data. Second, our sample was small and restricted to young adolescents. Future studies should replicate these results and test the association between emotion recognition skills and students’ behaviors during bullying episodes in both younger children and older adolescents.

Despite these limitations, taken together, the findings of this study documented the significance of considering a basic skill, namely recognizing facial emotions, for understanding different behaviors during bullying episodes. To date, this is the first study to offer a global picture on the association between these two variables and it aims to represent a basis for future studies. Indeed, several new research questions can arise from the current results. For example, it would be interesting to investigate which individual and contextual variables may mediate or moderate the relation between the individual’s ability of recognizing a specific emotion and his/her behavior and may, at the same time, help distinguish among different behaviors. Moreover, future studies could explicitly investigate the relations between emotion recognition and empathy as precursors of defending behavior; for example they could test whether the hypothesis about a possible direct link between better recognition of sadness and higher empathic concern is warranted. Furthermore, knowing which emotion students identify when they fail in recognizing the correct one (that is, what kind of “error” they do) may provide new insight on the relation between emotion recognition and students’ behavior.

## Supporting information

S1 Appendix Peer nominations on behavior during bullying episodes: List of items. (DOCX)
