# Supplementary material for: Associations between facial emotion recognition and young adolescents’ behaviors in bullying
Source: PLoS One. 2017 Nov 13;12(11):e0188062. doi: 10.1371/journal.pone.0188062 (PMC5683572; doi:10.1371/journal.pone.0188062)
Supplement: S1 Appendix — (DOCX) [file pone.0188062.s001.docx]

**S1 Appendix. Peer nominations on behavior during bullying episodes: list of items**

Participants were asked to nominate classmates who most often engaged in the behavior described in each item.

Bullying:

- is aggressive towards classmates, hit or push some of them;

- tease some classmates, calling them nasty nicknames, threatening or offending them;

- exclude some classmates from the group or do something so that they are isolated;

- spread rumors about some classmates or say mean things about other students behind their back.

Victimization:

- some classmates spread nasty rumors about them or say mean things when they can't hear;

- are attacked hard, hit or pushed by some classmates;

- some classmates call them nasty nicknames, insult them or offend them;

- are excluded or isolated from the group.

Defending:

- help or comfort classmates who are excluded from the group and isolated;

- defend classmates who are targeted by gossip or false rumors that are said behind their back;

- defend the classmates who are hit or attacked hard;

- defend classmates who are threatened or offended.

Passive Bystanding:

- stand by and mind his/her own business when a classmate is hit or pushed;

- mind his/her own business when he/she hear nasty rumors or mean things said about other students behind their back;

- do nothing and don’t interfere if a classmate is teased or threatened;

- act as if nothing has happened if he/she knows that someone is excluded or isolated from the group.
